# *“It’s changing our lives, not for the better. It’s important that we have a say”.* The role of young people in informing public health and policy decisions about gambling marketing

**DOI:** 10.1186/s12889-024-19331-x

**Published:** 2024-07-26

**Authors:** Hannah Pitt, Simone McCarthy, Melanie Randle, Grace Arnot, Mike Daube, Samantha Thomas

**Affiliations:** 1 Institute for Health Transformation, Deakin University, 1 Gheringhap St, Geelong, VIC 3220 Australia; 2 Faculty of Business and Law, School of Business, University of Wollongong, Wollongong, Australia; 3 Faculty of Health Sciences, Curtin University, Perth, Australia; 4 Curtin School of Population Health, Curtin University, Perth, Australia

**Keywords:** Gambling, Children, Young people, Marketing, Commercial determinants of health, Youth engagement, Advocacy

## Abstract

**Background:**

Marketing has a significant impact on the normalisation of gambling for youth across the globe. This has included shaping positive attitudes towards gambling, as well as increasing the social and cultural acceptance of gambling – particularly aligned with valued activities such as sport. Because of this, public health experts argue that gambling marketing poses a significant risk to the health and wellbeing of youth. While young people are increasingly exposed to, and impacted by marketing for gambling products, they are rarely consulted about policy issues and options. This study aimed to explore young Australians’ perceptions of current policy responses to gambling advertising, whether they thought young people should be involved in discussions and decisions about gambling marketing regulations, and their perceptions of the duty of governments to protect young people from gambling industry marketing strategies.

**Methods:**

Qualitative focus groups (*n* = 22) were held with *n* = 64, 12–17 year olds in the Australian states of Victoria and New South Wales. Participants were asked to reflect on current gambling policies, particularly relating to marketing, what they thought should be done about gambling marketing, and if and how young people should be included in public health responses to gambling. An interpretivist ‘Big Q’ approach to reflexive thematic analysis was used.

**Results:**

Young people highlighted the need for more effective regulations around the content and frequency of gambling marketing. They also wanted to see more realistic representations of the negative impacts of gambling to counter persistent positive commercial marketing messages. Most thought that young people should be given an opportunity to have a say about responses to gambling due to their unique experiences. Participants identified mechanisms to increase young people’s engagement in decision making, such as direct lines of communication to different levels of government, involvement in research, and diversifying ways of engagement. Specific recommendations included more regulatory action such as bans on gambling advertising.

**Conclusions:**

Creating formal structures that facilitate the inclusion of young people’s perspectives in decisions made about gambling can result in more innovative and effective strategies to prevent the harms from gambling industry products, promotions, and practices.

## Introduction

### Marketing is central to the gambling industry’s business model

Driven by new technologies and sophisticated marketing strategies, the products and promotions of the gambling industry present a public health risk to children, young people, and future generations [1, 2]. Technological advancements have significantly expanded the types of gambling products that are available and accessible within the community, the reach and availability of these products, and the ways in which they are promoted [1]. There are many different types of gambling, and advertising for newer forms of gambling is more prolific than for others. For example, in Australia, an analysis by the Australian Communications and Media Authority found that while there were over one million gambling ads aired on free-to-air TV (metro and regional) and metropolitan radio from 1st May 2022 – 30th April 2023, over 50% (502,800 spots) of these advertisements were from online gambling providers [3].

Marketing is central to the gambling industry’s business model across the world [4–6], and forms part of the discursive power of the industry [7, 8]. Discursive power involves influencing the framing of policy issues and their solutions, as well as shaping the actors deemed legitimate within the policy process [9]. This includes using marketing techniques strategically to normalise the socio-cultural acceptance of new and existing gambling products [10, 11], stimulate consumer demand and build product loyalty [12, 13], create perceptions that gambling is a fun activity that is central to social connections [14], embed gambling within valued social activities such as sport [15–17], and frame gambling as an activity that can be engaged with safely if individuals are informed and responsible [18, 19]. The framing of gambling through marketing is endorsed, facilitated, and amplified by a range of organisations with vested interests in profiting from gambling – including media companies, sporting teams and codes, and at times governments – despite significant public health and community concern about the impact of marketing on children and young people [1].

### Young people’s exposure to gambling marketing in everyday environments

As the gambling landscape has changed, so has the way that young people are exposed to gambling marketing. Independent (i.e. not funded by the gambling industry or organisations funded by the gambling industry) research has demonstrated that young people see gambling products and marketing in a range of social, physical, and symbolic environments [20, 21]. This includes a range of physical and online settings – from shopping centres to social media sites [20–22]. In a study with Danish adolescents (12–16 years old), 43% of participants reported that they saw gambling advertisements at least once a day in the media or elsewhere [23]. Novel marketing strategies, which often use celebrities and influencers and are communicated through a range of social media platforms, capture the attention of young people, normalise products, build trust and legitimacy, and reduce perceptions of risk [22, 24]. These novel strategies are of course not unique to gambling, and have also been demonstrated as strategies used by a range of industries that are harmful to health, such as vaping [25]. As Boerman and colleagues [26] argue, one of the main problems associated with these novel strategies – such as influencer marketing – is that young people may not be able to distinguish between commercial and non-commercial content. Studies have demonstrated this in gambling, with research showing that young people may not always recognise these types of strategies as ‘marketing’ to promote or sell a gambling product [22, 24].

Most research in this area to date has focused on the volume and placement of gambling advertising in sporting environments [27], and the influence this may have on normalising gambling for children and young people [28]. Studies examining the extent and nature of gambling promotions within sporting contexts demonstrate the volume of different types of marketing strategies used in these settings, including commercial break advertising, social media communications, sponsorship announcements, stadium signage, and jersey logos [22, 29, 30]. There is also extensive evidence regarding how young people recall and interpret the gambling marketing they see during sport. This includes how they symbolically associate gambling messaging as being a normal part of sport and associated with sports fan loyalty [15, 17, 31].

### The impact of gambling marketing on young people

Reviews of academic literature about the impact of gambling marketing on children and young people conclude that gambling marketing has a ‘pervasive presence’ in the daily lives of young people and plays a central role in introducing young people to gambling [32]. From a young age, children can recall brand names, specific advertising appeal strategies such as humour, celebrities, detailed plot lines, and the colours of particular brands [24, 31, 33]. Some of these appeal strategies can lead young people to perceive gambling as a fun and exciting activity to do with friends, and an easy way to win money [17, 34, 35]. Inducements and promotions also influence young people’s perceptions of the risks associated with gambling [31], with young people reporting that marketing deals and promotions could encourage them to want to gamble in the future [36].

There is some evidence that marketing may also play a role in young people’s gambling behaviour. A study of Australian adolescents (12–17 years) found that, among other factors, the number of types of gambling advertisements seen in the last month was associated with gambling in the last month [37]. In a multi-national study of 15–25 year olds, consumer debt, online gambling community participation, online casino participation, and exposure to online pop-up advertisements had strong associations with ‘problem’ gambling [38]. Further, marketing does not just occur through commercial advertising. For example, researchers engaging in critical analyses of gambling industry funded education activities have found that these types of activities may reproduce personal responsibility discourses – including encouraging young people to control their impulses—thus shifting the responsibility for gambling harm to children, young people, and their families [19]. In a study investigating young people and gambling in Nigeria, the authors outlined the range of marketing strategies introduced by bookmakers aimed at encouraging and increasing participation in gambling [39]. They proposed that the range of innovative strategies developed by the gambling industry *“could explain the prevalence of underage gambling, female participation in gambling, and problematic gambling”* [39], pg. 60]. In a further example, a study of 8–17 year olds in Ghana found that children who had radio access were more likely to have participated in gambling than those without radio access [40]. The authors highlighted that radio is one of the main marketing channels for gambling advertising in Ghana, particularly for sports betting [40], pg. 10].

### Restrictions on gambling marketing to protect children and young people

Concerns about the impact of gambling marketing on young people have led to calls for comprehensive public health approaches to restrict or ban gambling advertising, promotions, and sponsorship – similar to those that protect children from the marketing tactics of other harmful commodity industries [41, 42]. Some countries such as Belgium and Spain have enacted strong laws that aim to restrict most gambling marketing, specifically to protect children [43, 44]. In 2022, the Belgium Gaming Commission announced regulations that would restrict gambling marketing from most places including TV, social media, on the street, and direct promotions through email and text, as well as increasing the legal gambling age to 21 and introducing deposit limits [45]. However, these regulations have not come without significant backlash from the gambling industry and those with vested interests, which resulted in some of the changes being scaled back [46]. Researchers have highlighted that in other countries such as those in sub-Saharan Africa there is very limited legislation designed to regulate gambling marketing in a digital age [47].

In Australia, regulatory approaches to protecting young people from gambling advertising focus on restricting the timing and placement of gambling advertising, including banning gambling advertising near schools and on public transport in some Australian states [48], and during children’s television viewing hours [49]. However, there is evidence to suggest that despite attempts to restrict gambling advertising, the gambling industry has circumvented regulations by increasing the volume of advertising during other timeslots or on other media channels to which young people are also exposed [50]. In 2023, an Australian Federal Parliamentary Inquiry into online gambling and its impacts on those experiencing gambling harm recommended that a tobacco style ban on gambling marketing should be implemented over a three-year period with a specific aim to protect the “*next generation of young Australians*” [42]. However, the Australian Federal government has at the time of writing not decided if it will implement these recommendations.

### Including children and young people in decisions about their health and wellbeing

Young people have a right to be involved in decisions about their future and there is growing recognition that public health professionals should provide opportunities and mechanisms that allow for this involvement [51]. Public health researchers and advocates have highlighted that the development of policies about harmful marketing should involve considering the suggestions and experiences of young people [52, 53]. This aligns with principles outlined in the Convention on the Rights of the Child, which recognises the right of young people to voice their opinions and highlights the importance of considering their perspectives when adults are making decisions that impact them [54].

In some areas of public health such as tobacco, climate change, and junk food, young people have been actively involved in advocating for policy reform of harmful industry practices [51, 55, 56]. Youth-led advocacy organisations such as Bite Back 2030, have facilitated and empowered young people to be involved in advocating to governments, building skills and capacity, and setting agendas and priority areas [57]. Research shows that young people are highly capable of discussing public health issues and presenting clear and considered recommendations regarding how governments and the public health community should respond [51, 58, 59]. However, there are still only limited formal opportunities for young people to participate in the policy decisions that are made about gambling, or to recommend how they would like to be engaged in these decisions.

The aim of this study was to gain insights into young Australians’ perceptions of current policy responses to gambling advertising, whether they thought young people should be involved in discussions and decisions about gambling marketing regulations, and the duty of governments to protect young people from gambling industry marketing strategies. Three research questions were used to guide the study:

1. What do young people think about current policy responses to gambling marketing and would they do anything differently?
2. How do young people want to be engaged in decisions made about public health responses to gambling marketing?
3. What are young people's expectations of the role of government in influencing the decisions that are made about the promotion of gambling to protect children?

## Methods

### Approach

This research was part of a broader interpretive qualitative investigation into young people’s perceptions about gambling advertising, promotion, and sponsorship. Most qualitative public health research is interpretive and experiential in its approach. This is because the aim is not to provide generalisable findings using representative samples, but rather to contextualise and understand people’s experiences in the contexts of their everyday lives. Braun and Clarke ([60], pg. 3) describe this as being “*centred on the exploration of participants’ subjective experiences and sense-making”*. Importantly this includes thinking about developing inclusive practices and opportunities so that people can share their opinions about areas of health that may be important to them [61]. One other study has been published from this data set looking at the impact of celebrity and influencer endorsement of gambling [24].

### Criteria for recruitment

Participants were required to be 12–17 years old, be residents of the Australian states of Victoria or New South Wales (NSW), have parental consent to participate, and be proficient in English. The specified age range (12–17 years) was chosen based on research indicating that this is the developmental stage when young people begin to understand marketing strategies and discern the persuasive intentions [62]. This may make them particularly vulnerable to gambling marketing, as understanding the intent does not mean that they will be able to “*resist the persuasive influence*” of the strategies and potentially positive messages that they are exposed to [63], pg. 1479]. The states of Victoria and NSW were chosen due to their gambling expenditure (NSW the highest expenditure in Australia and Victoria the third highest [64]) and slightly different gambling cultures. Purposive and convenience sampling methods were employed, with recruitment facilitated through parents. Various recruitment methods, including posting on social media and sharing with sporting and community groups, were utilised to disseminate advertising material. Parents of prospective participants contacted the research team directly to express interest. Snowball sampling was also employed as parents shared study details with others they believed might be interested. Parents of interested participants received a Plain Language Statement outlining the study details, and informed written consent was obtained before organising focus groups.

Prior to the commencement of focus groups, young people were informed that they could stop or pause at any time. They were also directed to confidential help services if they felt the need to discuss any issues arising from the interview. Information about the study was provided verbally to participants and their verbal consent was obtained before the focus groups commenced. They received a $50 gift voucher as a token of appreciation for their time.

Approval for the study was granted by the Deakin University Human Research Ethics Committee [2021–304].

### Data collection

Focus group discussions with 2–4 participants were conducted via Zoom. The number of participants in each group was kept small, as we have found that this enables a more robust discussion among participants because they can feel more comfortable with less people and have an equal opportunity and more time to respond to questions. Focus groups also allow participants to draw on their own experiences, while also co-creating data by engaging with other participants and the facilitator’s perspectives. Focus groups were audio recorded with participants' and their parents' permission and lasted up to 90 min.

The themes for investigation in the broader study included; social norms related to gambling for young people; gambling advertising, marketing, and sponsorship; celebrity endorsement of gambling; perceptions of the relationship between gambling and sport; and the responsibility of protecting young people from gambling advertising. The sections of the interview guide discussed in this paper related to young people's perceptions of policies and strategies aimed at protecting young people from gambling advertising. Topics covered in the discussion guide included the extent to which young people should be included in decisions about their exposure to gambling marketing, what could be done to protect young people from gambling marketing, views on current restrictions and regulations of gambling marketing on television and social media, ways of educating young people about the risks associated with gambling, and what they would say to the Prime Minister about the impact of gambling marketing on young people in Australia. Photo elicitation methods were incorporated to prompt discussion and co-create data and knowledge [65]. The facilitator shared images and videos during the focus groups, such as gambling advertisements, screen shots of celebrity and influencer endorsements, newspaper headlines demonstrating different gambling regulatory approaches, a gamble responsibly message, and experiences of gambling harm, and invited participants to comment on the materials used. This enabled a collaborative effort between the researcher and participants to discuss and theorise the meanings of the images presented [65]. Data collection continued until focus group data provided sufficient information power to answer the research questions [66, 67].

### Data interpretation

The data was analysed using a reflexive approach to thematic analysis [68]. We used a ‘Big Q’ approach, which embraces researcher subjectivity and interpretivist paradigms [69]. ‘Big Q’ approaches situate knowledge as partial and contextual, see researcher subjectivity as a strength, and contrast positivist approaches in which researchers aspire for objectivity and the minimisation of bias [69], pg. 2].

To initiate this analytical process, the researchers immersed themselves in the data by reviewing the Zoom transcripts generated automatically during recording, listening attentively to the focus group discussions, and refining the transcripts to rectify any inaccuracies. Detailed notes were taken throughout the familiarisation phase to develop a nuanced understanding of the content of the data. During this process, the researchers critically engaged with the data to develop a deep understanding of young people’s responses. This involved collaborative sessions whereby the team deliberated on interpretations, challenged any pre-existing assumptions, and scrutinised our own perspectives on the data. This rigorous approach contributed to the robustness and credibility of the research findings.

**Fig. 1:**
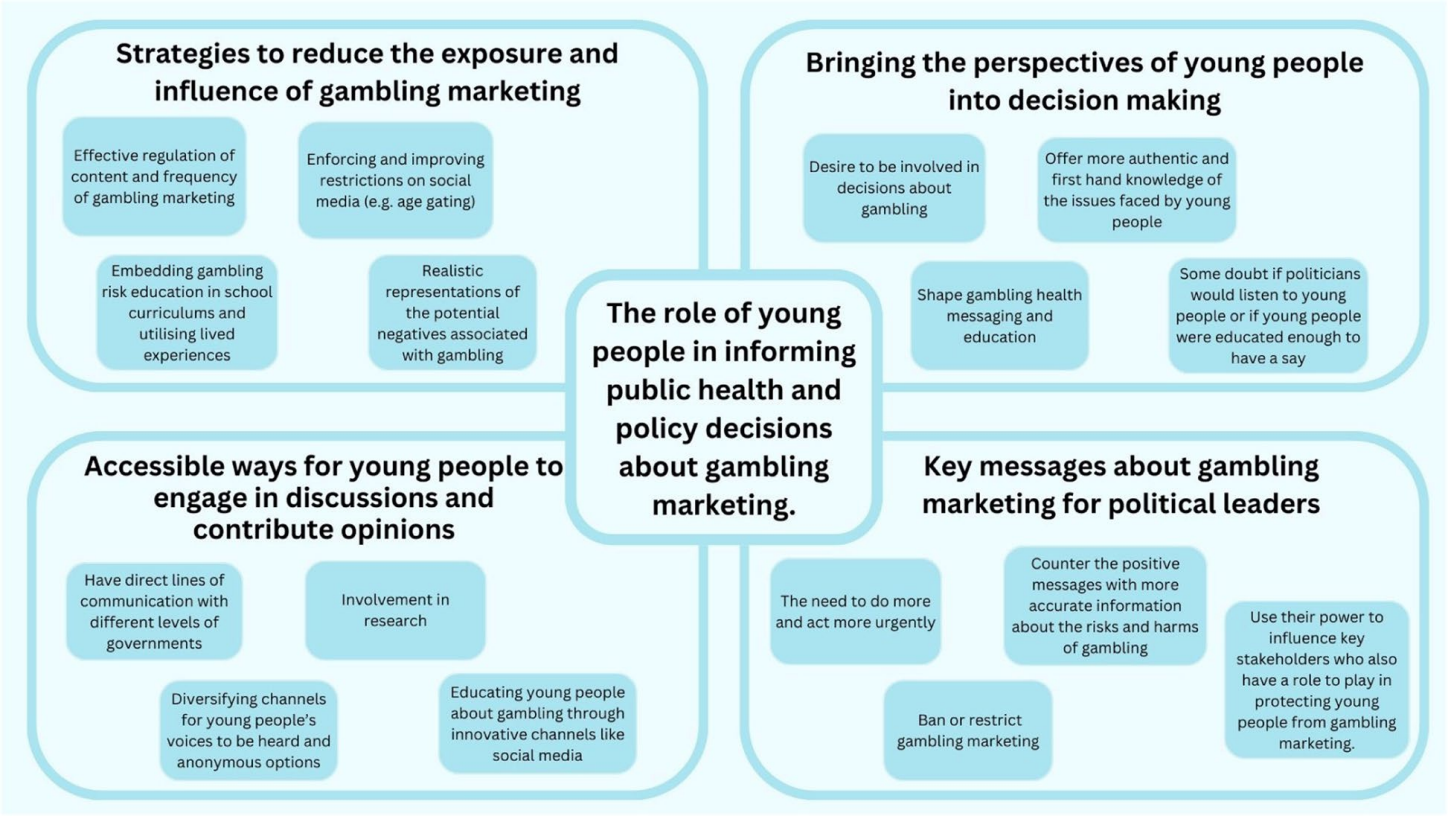
The role of young people in informing public health and policy decisions about gambling marketing

Data were managed through Microsoft Excel. Open coding techniques were applied to generate initial codes in line with the research questions, identifying both recurrent patterns and unique variations in participant responses. These initial codes were grouped into subthemes and overarching top-level themes were then refined, providing a comprehensive framework across the dataset. The analysis process occurred concurrently with data collection. This ensured that the interview schedule could be adapted in response to emerging ideas and themes. Regular team meetings were conducted to encourage discussions and reflections on the evolving interpretation of the data. During the write up of the results the theme names and content were refined, with themes reviewed to ensure they were distinct and addressed the research questions. Consistent with Braun and Clarke’s [70] approach, we did not assign numeric information for each idea presented in the themes to ensure that we were avoiding any “*positivist creep*” [71], pg. 2] and instead provided a selection of quotes to reflect our interpretations of the core meanings coming through from the data. The themes that were constructed from the data are shown in Fig. 1.

## Results

### Participant characteristics

Table 1 outlines the participant characteristics. The sample consisted of *n* = 64 young people aged 12–17 years across 22 focus groups. The focus groups were configured of 2–4 participants, with most focus groups containing three people (*n* = 14, 63.6%). There were nine focus groups of only boys (40.9%), nine mixed gender groups (40.9%), and four groups of only girls (18.2%). This resulted in two thirds of the sample being male (*n* = 44, 68.8%), and over half were aged 12–13 years (*n* = 33, 51.6%). Almost half of the focus groups were made up of Victorian only participants (*n* = 10; 45.5%), followed by focus groups of both Victorian and NSW participants (*n* = 8; 36.4%). Over half of the participants lived in Victoria (*n* = 38, 59.4%) and, based on SEIFA scores, the majority of participants were from middle to high areas of socio-economic advantage (*n* = 60, 93.8%).

**Table 1:** Participant characteristics (*n* = 64)

	n	%
**Age**		
12–13 years	33	51.6
14–15 years	21	32.8
16–17 years	10	15.6
**Gender**		
Male	44	68.8
Female	19	29.7
Non-binary	1	1.6
**State of residence**		
Victoria	38	59.4
New South Wales	26	40.6
**Socio-economic area**		
Low (score 1–3)	4	6.3
Middle (score 4–7)	17	26.6
High (score 8–10)	43	67.2

There were four key themes that were constructed from the data. The themes and subthemes from the data are represented in Fig. 1.

### Strategies to reduce the exposure and influence of gambling marketing

Young people highlighted the need for more effective regulation that addresses the content and frequency of gambling marketing because of the excessive exposure and the potential negative impact this may have on young people. There was a belief that exposure to gambling marketing, especially during the formative years, could shape young people’s attitudes and decisions related to gambling. For example, one 14-year-old girl stated that young people’s minds were *“very vulnerable”* to the influence of marketing and she believed this to be *“quite dangerous”.* Participants pointed out that current restrictions on gambling marketing on television, whereby gambling marketing was permitted after 7 pm, were insufficient because many young people stay up later than that. Young people reflected on their own bedtimes and called the 7 pm regulations *“outdated”*. Participants suggested a need to extend time restrictions to better align with the actual viewing habits of young people today.

The discussion extended to the importance of age verification mechanisms on social media, especially on platforms like YouTube where users could input their age, to ensure that only individuals above a certain age receive gambling-related marketing content. Participants criticised current age restrictions and rules governing gambling marketing, noting that some young people misrepresented their age on social media platforms. Some mentioned seeing gambling marketing content on social media despite having their age inputted correctly as under 18. Some participants suggested that the most effective way to protect young people would be to remove gambling marketing all together:

Specific gambling ads, like on Snapchat I get gambling ads. On games that you have to put your age in, I get gambling ads like a lot. I think they should be banned, get rid of them because they don't have a good impact at all. – 13 year old girl, Victoria.

Participants noted that young people are often only exposed to the positive sides of gambling without sufficient information about the downsides and risks involved. They felt that the current marketing landscape placed an emphasis on winning and positive outcomes, which created a distorted view of the risks of gambling. Young people suggested making marketing messages less appealing and more reflective of the potential negative consequences:

My advice is just putting the negatives in the ads or just not having the ads at all. – 13 year old girl, NSW.

Others suggested that governments could do more to raise awareness of the issues by creating gambling messages that countered the positive messages. Participants highlighted the importance of educating young people about the financial risks and negative consequences, emphasising that awareness of potential negative impacts might deter excessive gambling:

Seeing like an actual image of what like effects could actually happen to you personally. Say if you see someone just there having a hard time and you say, “This is after so and so has gone too far and tried to risk it all on a bet.” And saying like showing the actual possibilities of what could actually happen. And just, just to get in people’s head, “Oh no, that could actually happen to me. I’ll start taking better care and more consideration in what I’m – how much I’m gambling and all that. – 15 year old boy, NSW.

Young people suggested embedding education about gambling in the school curriculum *“from a young age”*. They believed that early exposure to the potential negative aspects of gambling could contribute to building a strong understanding of the risks of gambling. They emphasised the effectiveness of using real-life stories, including from individuals who had recovered from gambling addiction. They believed that personal narratives could make the educational content more impactful for students:

I think like in a school they could have a speaker come in [and] give the whole school a talk about why they shouldn't gamble. Because they can show them as many videos as they want, but like if they have somebody who has probably felt the effects of gambling right there, they will probably not gamble. – 12 year old boy, Victoria.

### Bringing the perspectives of young people into decision making

Young people expressed an interest in being involved in decision making processes related to gambling, emphasising the need for their voices to be heard. Participants highlighted that they are the ones directly influenced by gambling advertisements, and believed that their insights could provide a more accurate understanding of the impact on people their age:

We are the young people that they are influencing, and getting our opinion and our insight instead of adults trying to guess what we know. – 14 year old girl, Victoria.

Participants believed that involving young people in discussions and decision making would offer a more authentic perspective, as opposed to adults making assumptions about their experiences in relation to gambling. They indicated a desire to contribute to decisions aimed at protecting young people from the potential harms of gambling, because they had firsthand knowledge of the issues and challenges they faced in the current media landscape:

Teenagers watch TV, they see the ads. Like over 70% of teenagers follow sport, play a sport. Kids play sport. It’s very known to us and very out there. So, you know, if they were asking us when we were watching TV, what we were watching, when do we look at that? When do we look at this? Stuff like that. If they were involving us, trying to protect us, it would be better. But they don’t care. Like it’s, it comes down to the care, I don’t think they care. – 15 year old girl, NSW.

It's affecting us more than it's affecting, you know, you. It’s changing our lives, not for the better. It's important that we have a say. – 13 year old girl, Victoria.

Young people also felt that they should have a voice in shaping gambling messaging and education. They commented on the power of peer influence and relatability, suggesting that young people should be involved in conveying messages about the dangers of gambling to their peers. This approach was seen as more impactful than relying solely on older people to deliver the message. Participants believed that young people's perspectives were crucial in designing educational content, as they can offer unique insights and connect more effectively with their peers. Incorporating young voices into anti-gambling marketing was suggested:

If I saw on TV [someone] telling me not to gamble and they were like an older person, maybe middle aged or older, man or woman, versus a person my age telling me not to gamble and why, I would most likely pay more attention to the one with my age telling me. – 15 year old girl, NSW.

However, some young people expressed doubt about including very young people in these decisions. They considered that some children were too young (under 10 years old), and they might not comprehend the complexities of gambling-related decisions. Some believed that young people were already sufficiently engaged in decision making around gambling marketing. They considered that voting in political elections, which is required from age 18 in Australia, is an appropriate way and age for people to contribute to policy decisions. Other young people believed that decision makers would not consider young people’s opinions or take them seriously due to their age:

I still think people probably won’t listen to them because they're younger generations. – 13 year old boy, NSW.

### Accessible ways for young people to engage in discussions and contribute opinions

Participants suggested mechanisms to engage young people in decisions made about gambling marketing. Some suggested direct lines of communication with governments as a way of expressing concerns and proposing solutions. For example, a 14 year old boy proposed assemblies within schools involving parliamentarians. He envisioned a scenario where representatives from each class would attend meetings with key decision makers, providing a platform for sharing students' opinions on various matters, including gambling marketing. Another participant discussed the idea of integrating younger people into government teams and boards. This was seen as a way to making decision-making bodies more reflective of diverse age ranges, employing younger people who could bring fresh perspectives of younger people who were navigating modern society:

If they get younger people on like government teams. ‘Cause generally I find its older people that are on - it's definitely generally older people. So I think that the people that are working with the government should be able to hire 30 year olds, and younger than 30 year olds. They need to be able to do that to, so that younger people can share their experiences and to help. Because they generally have, they’ve grown up in the current world so they can understand it easier than older. – 15 year old boy, NSW.

Participants appreciated being asked their opinions in the current study and stressed that research aimed at understanding the younger generation's perceptions of gambling could contribute effectively to policy decisions. They claimed that focusing solely on adults might overlook valuable insights and innovative ideas. Young people also discussed the importance of diversifying channels for young people’s voices to be heard and considered. They suggested that creating different avenues for young people to voice their opinions anonymously could provide valuable information from those who otherwise might be less comfortable talking directly with decision makers:

We could probably involve young people more just by like putting a way out there for them to voice their opinions even if it’s like anonymous, an option to be anonymous. And just a way that young people can voice their opinions and that those opinions can have an impact. – 13 year old boy, NSW.

Participants believed that, given the right knowledge and information, young people could contribute meaningfully to decisions regarding gambling-related issues. Young people considered that one way to do this was to utilise social media, particularly platforms like TikTok, to engage their peers on the issue of gambling. They believed that such social media platforms could effectively communicate messages about the dangers and potential consequences of gambling, especially given their popularity among young people. Participants suggested creating impactful videos that highlighted personal stories, losses, and recovery journeys to resonate with audiences:

I reckon advertising or showing videos of people losing money, or showing how they recovered. Or showing people who were addicted to gambling and how they stopped, showing like their recovery story or showing how much money they lost would help a lot. Younger audiences could go “Yes, I could win $10,000 but I’m going to probably lose more. – 12 year old boy, Victoria.

### Key messages about gambling marketing for political leaders

When participants were asked what they would say to the Prime Minister about gambling marketing and young people, participants articulated clear and concise messages about the need to “*do more*”. They questioned the lack of decisions about gambling from the Prime Minister and his government, and the negative impact that this lack of action may have on future generations. There was a level of frustration from some participants that the government was not acting quickly enough to address gambling marketing, and that its complacency about gambling marketing and unwillingness to take decisive action were negatively impacting Australian society:

Do something about it before it gets worse. – 16 year old boy, Victoria.

Be more serious about it [gambling marketing], I guess, he’s kind of just letting it slide. – 16 year old boy, NSW.

He’s supposed to be responsible for having a good country but we’re making a bad country because of gambling. – 12 year old boy, NSW.

Some participants stated they would like to ask the Prime Minister to ban or restrict gambling marketing. They wanted the Prime Minister to know that positive gambling messages were contributing to the overall harm that was occurring in communities, normalising gambling, and encouraging adults and young people to want to gamble. One participant wanted to convey to the Prime Minister that it was not a surprise that people were gambling so much when all the messages were positive:

There’s so many ads saying yes. Why are you surprised that there’s so many people [gambling] and now you’ve come into this mess, where a lot of people are [gambling], because there's nothing saying no. It's like saying yes, yes, yes it's just peer pressure all the time. – 14 year old boy, NSW.

To counter these positive messages, participants wanted to tell the Prime Minister to ensure that there were more genuine representations of the risks associated with gambling, the gambling industry, and gambling promotions. However, there were also a few participants who thought that the Prime Minister needed his own education on gambling so that he was more aware of the real-life impact that gambling was having on communities. Some young people felt that the Prime Minister was disconnected from the reality of everyday life and the impact that gambling was having:

I would like [the Prime Minister] to know that we want the more negative impacts shown instead of just [the positive]. …. We don’t want it [harm prevention messages] washed over, we want it actually done in a meaningful way, so it has an impact. – 13 year old boy, NSW.

Maybe like just encourage [the Prime Minister] to actually go and see all the aspects of gambling for himself. Coz like people can tell him lots of things, but if you never see that, like, he won't really understand that. So, like, I would just encourage [the Prime Minister] to get out there more and kind of get to know, like more about gambling in our community. Because I feel like he's always like in Parliament and never really gets out and never really sees what it looks like outside of that. – 16 year old girl, NSW.

They also discussed a range of mechanisms that the Prime Minister could use to change attitudes towards gambling. For example, participants suggested that he could influence sporting organisations and social media providers to ensure that young people were not exposed to advertising:

[The Prime Minister] needs to put more pressure on social media and media networks and stuff to make sure that children aren't exposed to gambling ads, and ads for things that can harm them. – 16 year old girl, Victoria.

Finally, young people stressed the importance of the Prime Minister having a duty to care for the health and wellbeing of Australians and prioritise people over profits:

The wellbeing of the population is more important than the revenue that comes in from these sorts of businesses. – 15 year old boy, NSW.

## Discussion

**Fig. 2:**
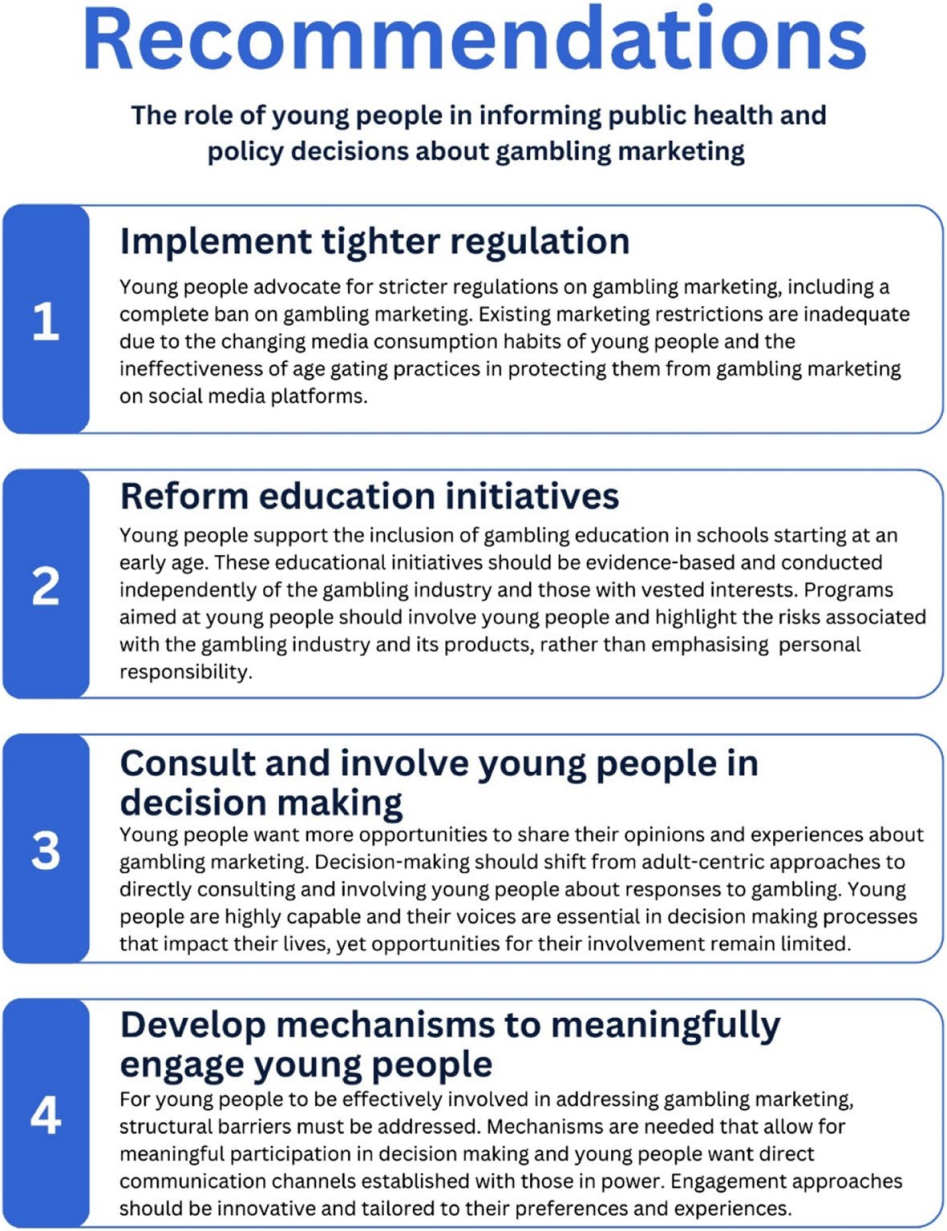
Recommendations for engaging young people in public health responses to gambling marketing

There is limited research that aims to understand whether young people are confident that governments and policy makers are responding adequately to the marketing tactics of the gambling industry, and whether young people are being engaged in discussions about the public health responses and policy decisions that are made about gambling marketing and are aware of the government actors that could have a powerful influence in creating policy change. Therefore, this study aimed to gain insights from young Australians about their perceptions of current policy responses to gambling advertising, whether they thought that young people should be involved in discussions and decisions about gambling marketing regulations, and the duty of governments to protect young people from gambling industry marketing strategies. There are three areas for discussion that relate to the research questions. A summary of the key recommendations is presented in Fig. 2.

Findings from this research suggests that young people—like many other public health and related stakeholders [42, 72]—are not confident that the decisions being made by policy makers about gambling marketing are effectively protecting children. Their discussions about current policies and regulations relating to gambling marketing revealed a range of perceived limitations. While current regulations restrict the timing and placement of gambling marketing messages [50], young people expressed concerns about the pervasive nature of gambling marketing across various media platforms, including social media. They viewed current regulations as outdated and insufficient, particularly given the evolving media consumption habits of young people, mainly via social media platforms, which is consistent with the findings of other studies [21, 73]. Young people were particularly concerned about the lack of regulations associated with social media platforms, with current policies (such as age gating) considered inadequate to prevent the exposure of young people to gambling marketing. Research from other areas of public health has also found age restriction controls to be ineffective in preventing children from accessing harmful industry content (such as alcohol marketing) on social media [74]. There is no reason to believe that gambling marketing messages would be any different. The present study adds to the growing body of evidence that young people support tighter regulation of gambling marketing, including outright banning of gambling marketing messages [75].

Participants in this study emphasised the importance of raising awareness about the risks of gambling for young people. They discussed the need to counterframe gambling marketing strategies, and suggested this could be done with campaigns that featured young people encouraging other young people not to gamble. These suggestions are very different from current education initiatives targeting young people (often funded by the gambling industry and legitimised by governments) that focus on personal responsibility paradigms of gambling harm minimisation [19]. In addition, young people proposed initiatives to enhance education and awareness about gambling-related harms, advocating for the inclusion of gambling education in schools from a young age. While education programs are an important part of a comprehensive public health approach to gambling harm prevention, they must be evidence based and delivered independently from the gambling industry and those with vested interests in the industry. Researchers have shown the significant issues that can arise when the gambling industry, or organisations funded by the gambling industry, are involved in developing and delivering youth education programs [19, 76].

Young people felt that they had important contributions to make, based on their own experiences and opinions, about the decisions that are made about gambling marketing. They stressed the importance of moving away from adult-centric approaches, to directly engage young people in discussions about gambling. Like other areas of public health concern, such as the climate crisis, young people have significant awareness of the strategies harmful industries are using to promote their products, and ideas about how to protect young people from the harms they may cause. Young people recognised that current policies and regulations are based around adult assumptions and entail approaches that limit young people from ‘having a say’. We would argue that this also extends to public health research which often collects data from young people about what they see in gambling marketing, but has rarely consulted them about what should be done. The young people in this study argued that they should be consulted and involved in decision making. This was because their demographic group is being directly influenced by gambling marketing, and because decisions being made by governments about gambling marketing are likely to have intergenerational consequences.

According to the principles outlined in international frameworks such as the Convention on the Rights of the Child (CRC), the voices of young people are essential in decision making processes that impact their lives [54]. Despite this recognition and many governments including Australia being a signatory to the CRC, opportunities for young people to participate in decisions related to gambling remain limited. This highlights a significant gap in current public health and policy practices.

Overcoming structural barriers to meaningful engagement is essential to realise the full potential of youth involvement in shaping policies and programs related to gambling and other public health issues related to the commercial determinants of health [51]. Young people highlighted the importance of direct communication channels, diverse representation (including in government departments), and innovative approaches tailored to their preferences and experiences. This includes mechanisms to bridge the gap between young people and people in positions of influence and power. Youth-led advocacy organisations Bite Back 2030 (addressing food systems) and the Campaign for Tobacco Free Kids (tobacco control) [77, 78] are focused on providing opportunities for young people to advocate for policy change. They have done this through enabling young people to share their experiences and solutions directly with decision makers. For example, in 2024 young people were invited to share their insights and experiences with the UK House of Lords Food, Diet and Obesity Committee [79]. The US Campaign for Tobacco Free Kids has a Youth Ambassador program that provides “*comprehensive advocacy training, mentorship and opportunities…and helps youth foster strong relationships with peers, their community and their legislators*” [80]. While there are some effective initiatives that enable young people to meet with politicians to discuss policy issues impacting them, researchers have criticised the lack of meaningful involvement of young people in formal decision-making processes [51, 58, 81]. Consistent with a public health approach, it is important that any strategies or initiatives that are developed are free from industry funding and involvement. By incorporating mechanisms to engage young people, policymakers can ensure that policies and interventions are more reflective of the needs, experiences, and perspectives of young people. This is important in empowering young people and creating a culture whereby young people’s perspectives about gambling are valued and integrated into decision making. Ultimately, this will enhance the effectiveness of initiatives that seek to addressing the risks associated with young people’s exposure to gambling marketing.

### Limitations

Participants in this study were young people from Australia’s two most populous states – NSW and Victoria. Different geographical areas may have different cultures associated with gambling products which may influence young people’s gambling attitudes. For example, unlike other states and territories, the state of Western Australia does not have gaming machines in the community. There were more boys than girls in the sample. This may suggest that parents see gambling as an issue that impacts males more than females. Given latest research that shows that young women are increasingly targeted by the gambling industry, researchers should make particular efforts to ensure that girls are invited into studies about the gambling and gambling marketing. This paper did not look to explore social factors that might be influencing young people’s attitudes towards gambling marketing and responses, such as parental gambling attitudes or behaviours.

## Conclusions

Findings from this study demonstrate that young people are aware of gambling marketing and have creative ideas about what should be done by governments and policy makers to minimise the negative impact of gambling marketing on children and young people. Creating formal structures that facilitate the inclusion of young people’s perspectives in decisions made about gambling will result in more innovative and effective strategies to prevent the harms associated with the gambling industry, and their products, and promotions.

## Data Availability

The datasets generated and/or analysed during the current study are not publicly available due to ethical reasons but are available from the corresponding author on reasonable request.

## Abbreviations

NSW: New South Wales
SEIFA: Socio-Economic Indexes for Areas
